# Electrocrystallization of Calcium Oxalate Mediated by Electrospun Polymer Fiber Using Poly(acrylic acid-*co*-4-styrene sulfonate)

**DOI:** 10.3390/polym17212888

**Published:** 2025-10-29

**Authors:** Andrónico Neira-Carrillo, Eddie Nieto, Nicole Butto-Miranda, Dania Cataldo, Bruno F. Urbano, Mehrdad Yazdani-Pedram

**Affiliations:** 1 Department of Biological and Animal Sciences, Faculty of Veterinary and Animal Sciences, University of Chile, Santiago 8820808, Chile; eddienieto.g@gmail.com (E.N.); nbutto@veterinaria.uchile.cl (N.B.-M.); dania.cataldo2@gmail.com (D.C.); 2 Department of Polymer Chemistry, Faculty of Chemical Science, University of Concepción, Concepción 3349001, Chile; burbano@udec.cl; 3 Department of Organic and Physical Chemistry, University of Chile, Santiago 8380544, Chile; myazdani@uchile.cl

**Keywords:** calcium oxalate, copolymers, electrospinning, crystallization, polymer fibers

## Abstract

Calcium oxalate (CaOx) crystals play a central role in urolithiasis, a pathological crystallization process that remains difficult to prevent. In this study, electrospun polymeric fiber (EPF) meshes of poly(acrylic acid-*co*-styrene sulfonate) P(AA-*co*-SS) were fabricated by electrospinning (ES) under controlled positive (+) or negative (−) voltages. The influence of PAA and PSS homopolymers, as well as P(AA-*co*-SS) copolymers with varying compositions, was evaluated as anionic scaffolds in in vitro CaOx electrocrystallization (EC) experiments. The structural and morphological features of the EPF meshes were characterized by scanning electron microscopy with energy-dispersive spectroscopy (SEM-EDS), Fourier-transform infrared spectroscopy (FTIR), and X-ray diffraction (XRD). Our results demonstrate that specific EPF meshes can effectively guide CaOx crystal growth, promoting the selective stabilization of either calcium oxalate monohydrate (COM) or calcium oxalate dihydrate (COD) phases. These findings highlight the potential of tailored EPF meshes as anionic scaffolds for modulating pathological CaOx crystallization.

## 1. Introduction

CaOx-type stones are among the most prevalent in urolithiasis. Several studies have ranked them as the main urolith worldwide [1,2,3,4], being the struvite the second most prevalent [5,6,7]. Clinically, CaOx occurs in two hydrated crystalline forms, CaOx monohydrate (COM) and CaOx dihydrate (COD), with COM being thermodynamically the most stable [8,9]. There is also the trihydrated crystalline form (COT), which is very unstable and scarce, being found in some plants [10]. Regarding the morphology, COM crystal has an anvil-like morphology while COD has a square-based bipyramid and COT has a prism shape [11,12]. Studies on urolith formation have focused on using biomolecules as modulatory agents to promote, inhibit or delay the nucleation and crystalline growth of CaOx [13,14,15,16]. It is known that urinary molecules can interact with calcium (Ca^2+^) and/or oxalate (Ox^2−^) ions. This interaction has been shown to induce the inhibition of CaOx nucleation and crystallization. The importance of the anionic charge of these inhibitory biomolecules has been reported [17,18]. For instance, sodium citrate bind Ca^2+^ forming calcium-citrate complex, thus making it unavailable for the formation of CaOx [19]. The prevention and treatment of CaOx stone formation can be aided by citrate [20,21]. Consequently, the presence of anionic urinary macromolecules is believed to hinder pathological COM formation through electrostatic interactions with Ca^2+^ ions. In contrast, weakly anionic macromolecules facilitate COM formation [22,23,24,25,26]. It is known that urinary calculi are formed in vivo on anionic fibrillar proteins mainly composed of aspartic acid and glutamic amino acids [27]. For this reason, the use of macromolecules with anionic functionalities are excellent candidates to evaluate their role on the nucleation and the stabilization of COM and COD crystals [17,18,28]. Therefore, a 3D fibrillar polymeric matrix containing anionic functional groups could be used as an organic scaffold for in vitro CaOx crystallization. The type and composition of these charges could be important for controlling crystal growth and morphology, as well as stabilizing the crystal type [29,30].

Polyacrylic acid (PAA) has been used for the in vitro crystallization of CaOx, demonstrating its ability to control the nucleation of CaOx by stabilizing crystalline phases and amorphous CaOx precursors [31,32]. PAA was shown to favor the COD formation over COM due to the presence of carboxyl groups in its structure [33]. The inhibitory capabilities of PAA has been demonstrated not only for CaOx [29] but also for calcium carbonate crystallization [34,35]. For instance, PAA possesses similar characteristics to endogenous biomolecules that inhibit CaOx crystal adhesion and aggregation, demonstrated in in vivo rat assays [29,36,37].

On the other hand, PSS is an anionic synthetic polymer that has also been used to stabilize calcite particles, which is the most stable polymorph of calcium carbonate [38]. Thus, PSS oligomers bind strongly to the polar surface of the crystal, demonstrating the importance of the solvent in the binding geometry of PSS oligomers on crystalline materials [39]. In addition, studies have shown that anionic groups, such as the sulfonate groups in sulfonated polystyrene and sulfonated polymethylsiloxane, play a key role in regulating the nucleation of calcium oxalate and calcium carbonate crystals, respectively [40,41]. ES process enables the formation of topologically controlled fibrillar arrays by applying polarity control of applied voltage [42,43,44,45].

The ES enables the fabrication of fibers with controlled orientation (random or aligned) and precise regulation of surface charge through the application of positive or negative voltages [46,47,48,49]. The combination of negative functionalities in electrospun polymer fiber (EPF) meshes derived from PAA-*co*-PSS copolymers further expands their potential applications in the study of pathological processes, in the design of controlled-release systems, and in the development of novel biomimetic surfaces for evaluating antiurolithic therapies [50,51]. Then, EPF meshes constitute an excellent substrate for investigating pathological mineralization processes, as they allow the study of how the microenvironment influences the attraction or repulsion of ions interactions that determine the morphology and type of CaOx crystals [29]. Therefore, in vitro electrocrystallization (EC) offers a controlled and reproducible model that facilitates a detailed understanding of the role of surface charges of solid scaffolds in pathological mineralization [16,47,48]. The proposed strategy offers an innovative platform for studying urolith formation, with potential application for the therapeutic approaches in the clinical management of this pathological condition. Thus, EC not only yields crystals at the end of the mineralization process but also identifies the precise moment at which nucleation occurs [32]. EPF meshes fabricated via ES act as anionic scaffolds capable of modulating CaOx crystallization, positioning them as valuable experimental tools for biomineralization studies and the development of preventive strategies against urolithiasis [52,53]. Accordingly, in the present study we evaluated the effect of PAA, PSS homopolymers, and P(AA-*co*-SS) copolymer EPF meshes, fabricated by ES, as organic scaffolds on the in vitro EC of CaOx.

## 2. Materials and Methods

### 2.1. Reactans

Reagents of the highest available grade were used. All solutions were prepared using fresh Milli-Q distilled and bi-distilled water (Labostar^TM^ TWF, Evoqua Water Technologies LLC, Warrendale, PA, USA). Sodium 4-styrene sulfonate and acrylic acid (99%) for the P(AA-*co*-SS) synthesis and the ethanol and dimethylformamide (DMF) for the EPF meshes fabrications were purchased from Sigma-Aldrich (St. Louis, MO, USA). Ammonium persulfate (APS, 98%, Sigma-Aldrich) was used as the initiator for the radical polymerization of the P(AA-*co*-SS) sample.

### 2.2. Synthesis of Homo- and Copolymers

**Figure 1:**
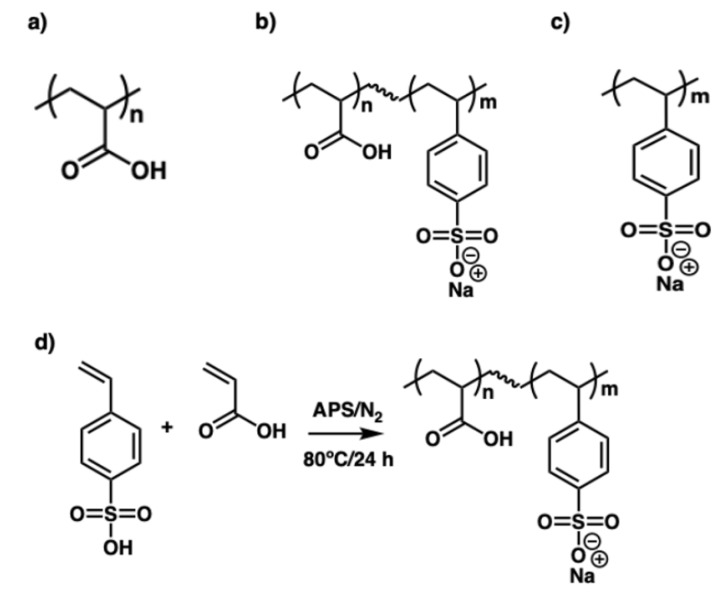
Chemical structures of (**a**) poly(acrylic acid) (PAA), (**b**) poly(acrylic acid-*co*-styrene sulfonate) P(AA-*co*-SS), (**c**) poly(styrene sulfonate) (PSS), and (**d**) the copolymerization reaction scheme.

The PAA and PSS homopolymer and P(AA-*co*-SS) copolymers were synthesized via free radical polymerization (Figure 1). Briefly, acrylic acid (AA) monomer was purified by removing the 4-methoxyphenol inhibitor using a packed column (Sigma-Aldrich, St. Louis, MO, USA). For the homopolymer synthesis, 0.11 moles of each monomer (AA or SS) were utilized. Copolymers with different AA:SS molar ratios (70:30, 50:50, and 30:70) were prepared in an aqueous solution using a 1.0 mol.-% APS initiator relative to the total monomer concentration. Polymerization reactions were conducted in 40 mL bi-distilled water under N_2_ atmosphere at 80 °C for 24 h. Purification process involved dissolution resulting polymer in 2000 mL of water, followed by sequential membrane fractionation using 10, 30, and 100 kDa molecular weight cutoff membranes (MWCOs). Then, copolymer was purified using a sequential ultrafiltration approach with progressively larger MWCOs. First, the sample was filtered through a 10 kDa membrane, retaining the >10 kDa fraction. This retentate was then fractionated using a 30 kDa membrane, isolating the >30 kDa fraction. Finally, the >30 kDa retentate was subjected to ultrafiltration with a 100 kDa membrane, yielding a copolymer sample with molecular weight ≥ 100 kDa. All purification and fractionation steps were carried out at room temperature using deionized water as the solvent.

**Figure 2:**
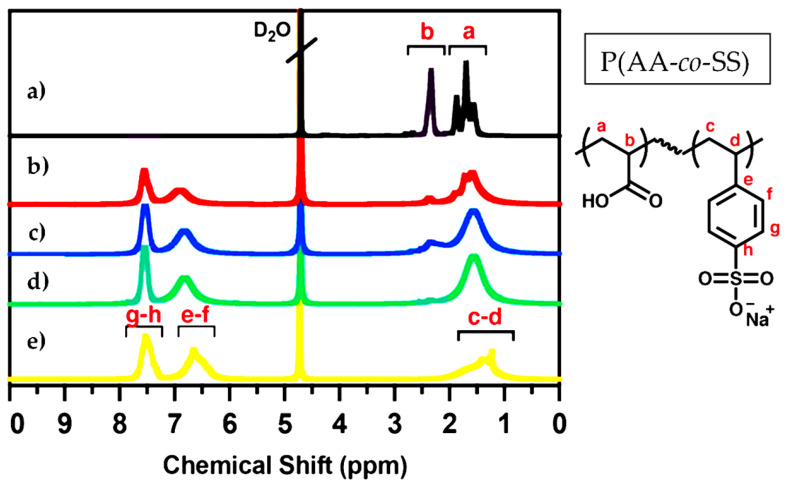
^1^H-NMR spectra of PAA, PSS and P(AA-*co*-SS) copolymers. (**a**) PAA, (**b**) P(AA-*co*-SS) (70:30), (**c**) P(AA-*co*-SS) (50:50), (**d**) P(AA-*co*-SS) (30:70), (**e**) PSSNa. Deuterium oxide (D_2_O) was used as the deuterated solvent. The ^1^H-NMR spectra reveal characteristic aliphatic and aromatic proton signals corresponding to the P(AA-*co*-SS) copolymers. The letters a–h denote the proton assignments in the chemical structure of P(AA-*co*-SS).

The PAA and PSS homopolymers and P(AA-*co*-SS) copolymers with different compositions were characterized by NMR technique to verify their structures (Figure 2). The ^1^H-NMR was obtained in an NMR spectrometer (Bruker Ascend 400 MHz) (Billerica, MA, USA) Deuterium oxide (D_2_O) was used as a deuterated solvent for NMR analysis. NMR analysis is a robust method for validating the chemical structure and purity of homo- and P(AA-*co*-SS) copolymer samples, as it confirms that they are free of initial monomers. The absence of signals attributed to vinyl protons at around 5.0–5.9 ppm in all NMR spectra indicates the absence of residual monomer impurities. In the ^1^H NMR spectrum of PAA, the signals at 1.1–2.1 ppm and 2.8–2.9 ppm are assigned to -CH_2_ and -CH, respectively [54]. It should be noted that the protons of the carboxyl groups were not observed due to chemical exchange. The ^1^H-NMR spectrum of PSS shows broad signals between 6.5 and 8.0 ppm, which are characteristic of aromatic protons. There are also signals in the range of 1.0–2.0 ppm, which are ascribed to the protons of the polymer’s main chain [55]. However, the ^1^H-NMR spectra of P(AA-*co*-SS) copolymers showed signals from both aromatic and aliphatic protons masked by the signals from PAA and PSS.

### 2.3. Preparation of EPF by ES

The ES technique was employed to fabricate EPF meshes from PAA and PSS homopolymers, as well as from P(AA-*co*-SS) copolymers with varying compositions, solvents, and processing parameters at 25 °C. P(AA-*co*-SS) copolymers with AA:SS ratios of 70:30, 50:50, and 30:70, along with the corresponding homopolymers, were electrospun into meshes with randomly oriented fibers using a flat-plate collector. EPF meshes with either positive or negative surface charge were obtained by applying positive (+) or negative (−) voltages in the range of 13–23.5 kV during the ES process.

For the preparation of EPF meshes on ITO substrates, the ITO was pre-mounted on the collector prior to ES. This arrangement allowed the polymer fibers to be deposited directly onto the ITO surface, thereby eliminating the need for any additional handling or transfer of the electrospun meshes. Each ITO substrate coated with a specific EPF mesh was subsequently employed in both the EC experiments and the various characterization analyses.

For the preparation of PAA mesh, an 8% (*w*/*v*) solution in a 1:1 water–ethanol mixture was used, while the PSS mesh was fabricated using a 20% (*w*/*v*) solution in a 2:1:1 DMF–water–ethanol mixture. For the preparation of the 30:70 P(AA-*co*-SS) mesh, a 25% (*w*/*v*) copolymer solution in a 2:1:1 mixture of DMF–water–ethanol was utilized. In the case of 70:30 and 50:50 P(AA-*co*-SS) meshes, 4% and 10% (*w*/*v*) copolymer solutions in 1:1 water–ethanol mixture were used, respectively. All polymer solutions were kept on an orbital shaker for 24 h, then stirred on a magnetic stirrer at 25 °C for 2 h, and passed through a Swinnex holder (Milli-pore Sigma^TM^, Merck, Darmstadt, Germany) as standard protocol for preparing EPF meshes. These solutions were then loaded into 5 mL luer-lock syringes (NIPRO^®^) and placed in the ES Fluidnatek^®^ LE-10 equipment to produce EPF meshes. The ES operation was carried out in Fluidnatek^®^ LE-10 instrument (Bioinicia SA, Valencia, Spain). The (+) or (−) applied voltage was selected by using bipolar high-voltage power supply accessory (from −30 kV to +30 kV) incorporated in ES equipment. Table 1 presents the ES parameters used for the preparation of each EPF mesh from PAA, PSS homopolymers, and P(AA-*co*-SS) copolymers. The EPF meshes were fabricated at a constant temperature of 25 °C, with flow rates ranging from 300 to 1200 µL/h and applied voltages between 13 and 23.5 kV. The distance between the aluminum-foil-covered collecting plate and the metal needle was set between 15 and 20 cm. Identical ES parameters were employed for both positive (+) and negative (−) voltages. Each EPF mesh was produced using 10 mL of the corresponding polymer solution.

### 2.4. In Vitro EC of CaOx

In vitro CaOx EC was performed in the presence of EPF meshes on indium-tin oxide (ITO) substrates in an electrochemical cell at 25 °C for 5 min using 9 mA. Additionally, control EC assays were performed using each homopolymer and copolymer samples as additives on the ITO surface or in the electrochemical solution (ECS).

CaOx EC assays were performed using an ITO with dimensions of 15 × 25 mm, which was placed directly on the collector plate. For the EC assays, the ITO glass was placed in an electrochemical cell containing a solution of 50 mM calcium nitrate and 75 mM ethylenediaminetetraacetic acid (EDTA). The pH was subsequently adjusted to 10.5 using 1 M NaOH added dropwise under constant stirring. This solution was then mixed with a 50 mM sodium oxalate solution and sonicated for 5 min. Finally, 25 mL of the resulting ECS was poured into the electrochemical cell.

For all CaOx EC experiments, homopolymer and copolymer EPF were deposited onto ITO glass surface, and then in vitro EC was performed. In the first set of control experiments, 300 µL of polymer solution were deposited on the ITO surface without EPF. Then, the solvent was evaporated until obtaining a polymer film. In the second set of control experiments, each polymer solution containing 15 mg of polymer was added to the ECS. In vitro EC was also performed for both control experiments. For the EC, an applied potential of ±10 V and 9 mA, with a sample interval of 2 s, was used in all cases. The electrochemical cell consisted of a three-electrode system comprising ITO-coated glass as the working electrode (WE), a coiled platinum (Pt) wire as the auxiliary electrode (AE), and the Ag/AgCl (3 M KCl) as the reference electrode (RE).

### 2.5. Characterization of EPF and CaOx Crystals

The morphologies of the CaOx crystals and the EPF meshes were analyzed using optical (OM) and scanning electron (SEM) microscopies with the LAZ morphometric program (Image Pro-Plus, Media Cybernetics, Melville, NY, USA) on a Nikon Eclipse E400^®^ and a JEOL JSM-IT300LV (JEOL USA Inc., Peabody, MA, USA) instruments, respectively. For the SEM analysis, the samples were coated with a 20 nm of gold layer by using a Denton Vacuum Desk V sputtering system in an argon atmosphere to ensure electrical conductivity. The SEM images were acquired using an accelerating voltage of 20 kV. The average diameter of the fibers and crystals was determined from collected SEM images using ImageJ software (NIH, USA; https://imagej.net/ij/). Microanalysis of EPF meshes was performed using energy-dispersive X-ray spectroscopy (EDS) with an AZtec Oxford detector coupled to the SEM equipment operated at 20 kV. Fourier transform infrared spectroscopy (FTIR/ATR) of CaOx and all homo- and copolymer P(AA-*co*-SS) EPF samples were analyzed by using an Interspec 200-X instrument (Interspectrum OU, Toravere, Estonia). X-ray diffraction (XRD) patterns of CaOx crystals grown on EPF meshes produced under positive (+) or negative (−) applied voltage were recorded using a D2 Phaser XRD system (Bruker, Karlsruhe, Germany) equipped with a Lynxeye detector and a Cu Kα radiation source (λ = 0.15406 nm) operating at 30 kV and 10 mA. A continuous scan was performed on an air-dried sample on a glass plate, with a step width of 0.02°, from 10 to 60° 2θ. Rietveld refinement analysis was performed to determine the relative phase composition of COM and COD in the XRD patterns of CaOx crystals obtained with the all EPF meshes under positive (+) and negative (−) voltages, using TOPAS version 4.2 (Bruker AXS, Karlsruhe, Germany) software.

## 3. Results

The surface charge of the PAA, PSS and P(PAA-*co*-PSS) copolymers EPF meshes were modified by applying a positive (+) or negative (−) voltage during the ES process. Figures S1 and S2 show the optical microscopy (OM) images of the meshes of PAA and PSS homopolymers and P(PAA-*co*-PSS) copolymers of different compositions (50:50; 70:30 and 30:70) and the OM images of CaOx crystals grown via EC on ITO substrate in the presence of homo- and copolymer EPF under positive (+) or negative (−) applied voltages, respectively.

**Figure 3:**
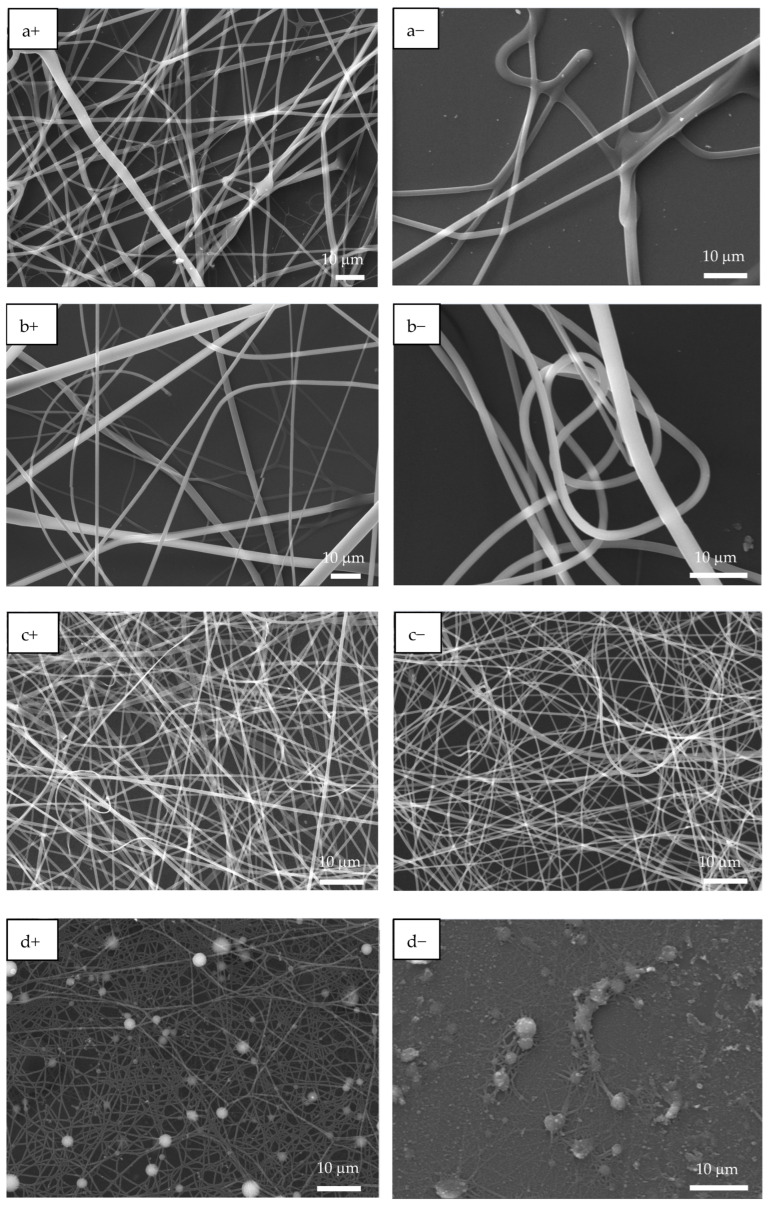

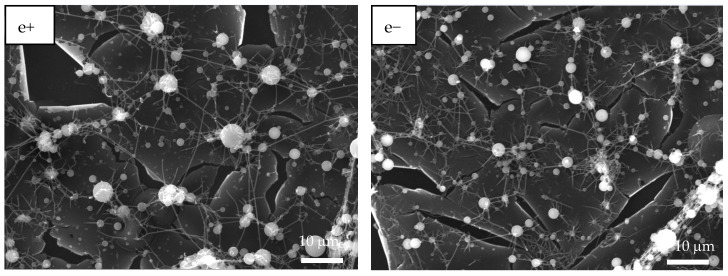
SEM images of EPF meshes produced from PAA, PSS and PAA:PSS under both positive (+) and negative (−) applied voltages. (**a+**,**a−**) PAA, (**b+**,**b−**), P(PAA-*co*-PSS) 70:30, (**c+**,**c−**) P(PAA-*co*-PSS) 50:50, (**d+**,**d−**) P(PAA-*co*-PSS) 30:70, and (**e+**,**e−**) PSS.

Figure 3 shows the SEM images of PAA and PSS homopolymers and P(PAA-*co*-PSS) copolymers with applied (+) or (−) voltages. The PAA homopolymer (Figure 3a+,a−) generated homogeneous fibers with defined edges and a smooth surface. The average sizes of the PAA fibers produced by (+) or (−) voltages were 1.6 ± 0.56 µm and 1.6 ± 0.74 µm, respectively. In the case of 70:30 P(PAA-*co*-PSS) copolymer produced homogeneous fibers with defined edges and a smooth surface with sizes of 2.1 ± 1.4 µm or 1.47 ± 0.76 µm at (+) or (−) voltages, respectively (Figure 3b+,b−). On the other hand, the 50:50 P(PAA-*co*-PSS) copolymer (Figure 3c+,c−) formed homogeneous fibers with defined edges and a smooth surface. The average size was 665 ± 185 nm with a (+) voltage and 665 ± 306 nm with a (−) voltage. In contrast, using the 30:70 P(PAA-*co*-PSS) copolymer, which contains a higher propotion of the SS monomer, formed beads and fibers with irregular edges and rough surface (Figure 3d+,d−). The fiber and bead sizes were 426 ± 96 nm and 1.9 ± 0.90 µm at a (+) voltage and 418 ± 84 nm and 1.84 ± 0.86 µm at a (−) voltage, respectively. When the PSS were used (Figure 3e+,e−), thinner heterogeneous fibers with abundant beads were observed. The presence of polymeric material was detected on the ITO surface. The average size of the PSS fibers was 313 ± 74 nm with the (+) voltage and 292 ± 63 nm with the (−) voltage. Bead sizes ranged from 1.36 ± 1.19 µm to 1.7 ± 0.96 µm with the (+) or (−) voltages, respectively.

While SEM provides morphological information, SEM-EDS confirms both the presence and the elemental composition of the electrospun fiber meshes on the ITO substrate, thereby supporting their chemical integrity. Then, EDS provided complementary elemental evidence of the scaffold composition and of the spatial distribution of Ca and O during CaOx crystallization. We performed SEM-EDS microanalysis on EPF samples of P(AA-*co*-SS) homopolymers and copolymers obtained on ITO substrates under both positive (+) and negative (−) voltages (Table 2). Table 2 shows the presence of sulfur (S) and sodium (Na) in the PSS homopolymer and in all P(AA-*co*-SS) copolymers. Furthermore, aluminum (Al), silicon (Si), indium (In), and tin (Sn) were detected, originating from the ITO substrate.

### 3.1. FTIR of Homo- and Copolymer P(AA-co-SS) EPF Fibers

FTIR was employed to verify that the EPFs used as scaffolds in the EC experiments retained their original functional chemical groups during the ES fabrication of the meshes.

**Figure 4:**
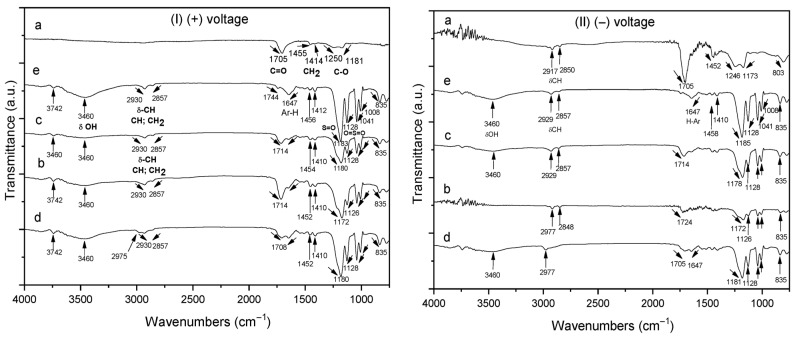
FTIR spectra of homo- and copolymer P(AA-*co*-SS) EPF meshes produced under positive (+) and negative (−) applied voltages. (**Ia**,**IIa**) PAA, (**Ib**,**IIb**), P(PAA-*co*-PSS) 70:30, (**Ic**,**IIc**) P(PAA-*co*-PSS) 50:50, (**Id**,**IId**) P(PAA-*co*-PSS) 30:70, and (**Ie**,**IIe**) PSS.

Figure 4(I) shows the FTIR spectra of homo- and copolymer P(AA-*co*-SS) EPF meshes produced under (+) applied voltage. Figure 4(Ia) shows the presence of the absorption bands of the carbonyl (C=O) of PAA at 1705 cm^−1^ and the methylene (CH_2_) groups at 1455 cm^−1^, respectively. The absorption bands at 1250 cm^−1^ and 1181 cm^−1^ were assigned to the stretching and tension (ν) modes of C-O, respectively. However, the absorption band of the hydroxyl (-OH) group of PAA at 3250 cm^−1^ was not observed due to the insignificant adsorption of the EPF meshes on ITO [56]. In the case of copolymer samples of different compositions, the FTIR spectra show the absorption bands corresponding to PAA and PSS homopolymers, where Figure 4(Ib) corresponds to 70:30 P(AA-*co*-SS), Figure 4(Ic) to 50:50 and Figure 4(Id) to 30:70 copolymers, respectively. As seen in Figure 4(Ib–d), the copolymers exhibited the same absorption bands as the homopolymers, although the intensity of certain bands shifted (e.g., the C=O and S=O bonds). For instance, the absorption band of the carbonyl group of PAA at 1705 cm^−1^ shifted from 1708 to 1714 cm^−1^ and the intensity of the stretching vibrations of the S=O and O=S=O bonds changed [56]. Figure 4(Ie) shows the asymmetric and symmetric C-H stretching vibrations at 2930 and 2857 cm^−1^ of the alkyl group of PSS, respectively. Additionally, stretching bands for the C=C bonds of the aromatic rings were observed at 1647 cm^−1^ and 1458 cm^−1^. On the other hand, stretching vibrations of the S=O and O=S=O bonds were observed at 1183 and 1128 cm^−1^, respectively, as well as the symmetric SO_2_ stretching at 1041 and 1008 cm^−1^ [57,58].

On the other hand, Figure 4(II) shows the FTIR spectra of the homo- and copolymer P(AA-*co*-SS) EPF meshes produced by applying a (−) voltage. The same features are generally observed as when a (+) voltage was applied, such as shifts in some absorption bands and changes in intensity. In addition, Figures S3 and S4 show a comparison of the different FTIR of the homopolymer meshes (Figure S3) and of the different copolymers (Figure S4) obtained at (+) or (−) voltages.

The observed shifts in the FTIR absorption bands of the PAA, PSS homopolymers, and the P(AA-*co*-SS) copolymer EPF meshes can be attributed to intermolecular interactions and changes in the chemical environment caused by fiber formation and copolymerization [59,60]. For PAA, the carbonyl stretching vibrations shift slightly due to hydrogen bonding between carboxyl groups within the PAA mesh, which alters the electron density around the carbonyl groups [61]. In the case of PSS, the shifts in the sulfonic acid group (-SO_3_H) and aromatic C=C stretching bands can result from ionic interactions between the negatively charged sulfonate groups and neighboring polymer chains, leading to modifications in the vibrational energy levels [62,63,64].

In the P(AA-*co*-SS) copolymer fibers, the FTIR spectra show combined features of both monomer units. The shifts in absorption bands compared to the respective homopolymers reflect additional inter- and intramolecular interactions, such as hydrogen bonding between carboxyl and sulfonate groups, electrostatic interactions, and potential changes in polymer chain conformation induced by the ES process. These interactions affect the local chemical environment of functional groups, resulting in the observed band shifts [65].

### 3.2. Chronopotentiometry Evaluation During CaOx EC Assays

**Figure 5:**
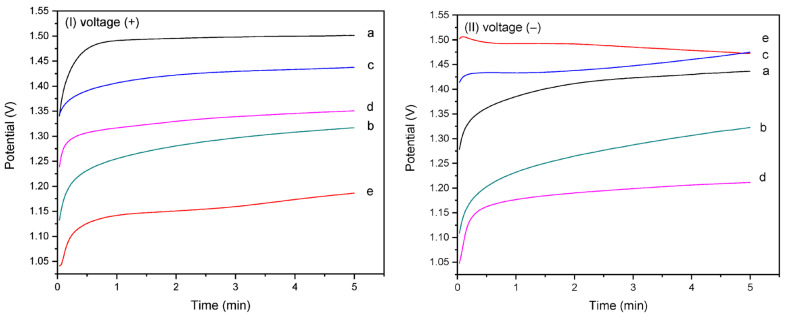
Chronoamperometric curves of homo- and copolymer P(AA-*co*-SS) EPF meshes during CaOx EC applying (+) (**I**) or (−) (**II**) voltages to: (a) PAA, (b), P(PAA-*co*-PSS) 70:30, (c) P(PAA-*co*-PSS) 50:50, (d) P(PAA-*co*-PSS) 30:70, and (e) PSS.

Figure 5(I,II) present the chronopotentiometric behavior of the homo- and copolymer EPF meshes on the CaOx EC under applied positive (+) or negative (−) voltages, respectively. The CaOx crystals formed on the ITO surface are non-conductive and act as barriers to oxygen diffusion, leading to a decrease in the measured potential (V), as shown in Figure 5. In Figure 5I, the potential (V) during CaOx EC is highest for PAA (Figure 5Ia). The P(AA-*co*-SS) copolymer meshes show intermediate potential values compared with the PAA and PSS homopolymers, as seen in Figure 5(Ib) (70:30), Figure 5(Ic) (50:50), and Figure 5(Id) (30:70). In contrast, the PSS mesh exhibits the lowest potential value during CaOx EC (Figure 5(Ie)).

Conversely, when a (−) voltage was applied, Figure 5II, an inversion of the potential (V) values was observed, being for PAA lower than PSS and 50:50 P(AA-*co*-SS) meshes (Figure 5(IIa)). The 50:50 P(PAA-*co*-PSS) copolymer exhibited intermediate potential (V) values between the two homopolymers (Figure 5(IIc)). However, an inversion of the potential values was observed for the 70:30 (Figure 5(IIb)) and 30:70 (Figure 5(IId)) copolymers. In contrast, the PSS mesh exhibited the highest potential value during CaOx EC (Figure 5(IIe)). The behavior of the potentiometric curves showed an increase in voltage with increasing resistance during the EC assay, showing a progressive stabilization of the potential curve as CaOx crystals are deposited on the ITO surface. In both CaOx EC experiments (Figure 5(I,II)), the EC showed an increase in voltage, indicating that the crystal growth occurred within 30–50 s for (+) voltage and 15–120 s for (−) voltage during the EC assay.

Moreover, Table 3 shows the initial and final potential (V) values recorded during the CE of CaOx for all EPF meshes and the additives (controls) under both (+) or (−) voltages. A negative potential (V) value in the potentiometric curves were observed for PSS with (−) voltage and for PAA used as an additive (control) in the CES, in which the final potential values were lower than the initial value during the EC. On the other hand, the lowest difference between the initial and final potential (V) values registered during the EC experiments was observed with the 50:50 P(PAA-*co*-PSS) mesh when using all EPF meshes for both (+) or (−) voltages.

Additionally, control CaOx EC experiments were performed using all EPF meshes as additives on the ITO surface (Figure S5) and in the ECS (Figure S6). It was found that the smallest difference between the initial and final potential (V) values was obtained with PSS and PAA homopolymers.

### 3.3. Scanning Electron Microscopy (SEM) Analysis

The presence of crystalline material after EC tests was preliminarily analyzed by optical microscopy. Although OM has limited resolution, different morphologies and sizes of CaOx crystals deposited on the ITO-coated substrate modified with homo- and copolymer meshes under (+) or (−) voltage were distinguished (Figure S2). At higher magnification, flower-like morphology characteristic of COD crystals was clearly identified. Distinct crystal types with different sizes and shapes were observed at both voltages. Thus, spherical, irregular, and square-based pyramid (COD) crystals with elongated projections were observed.

**Figure 6:**
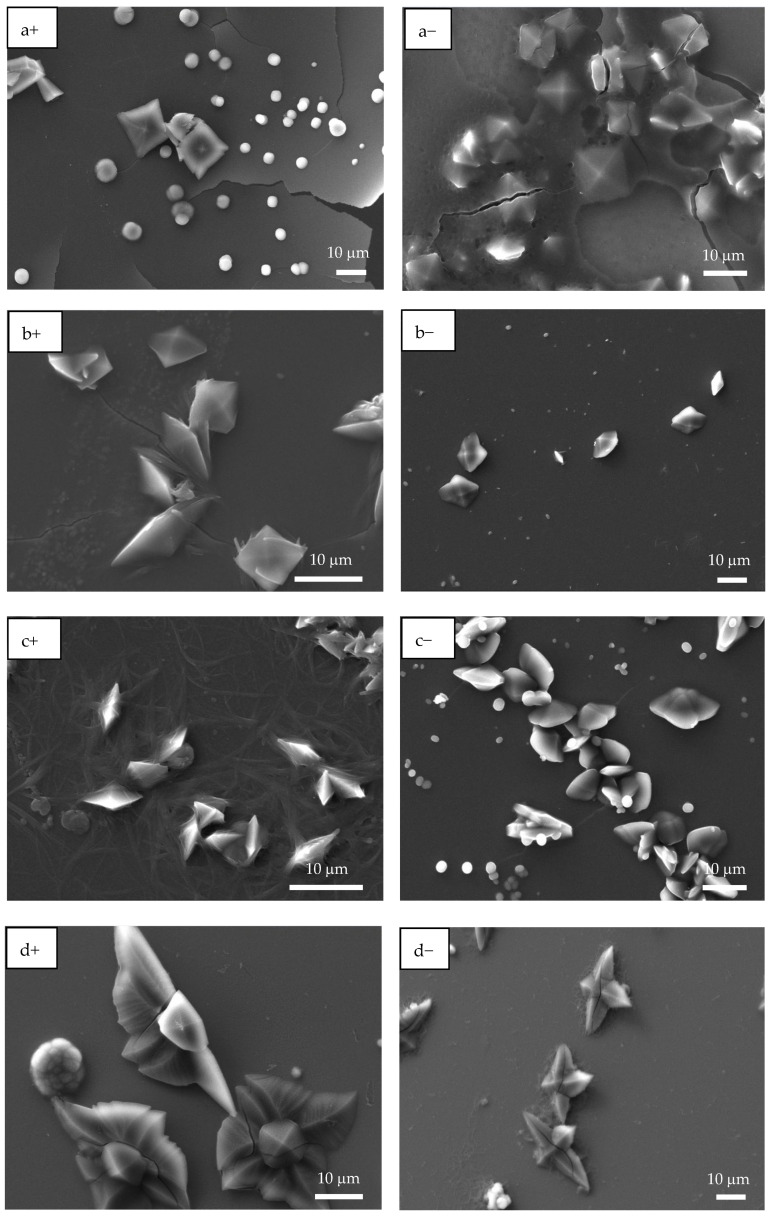

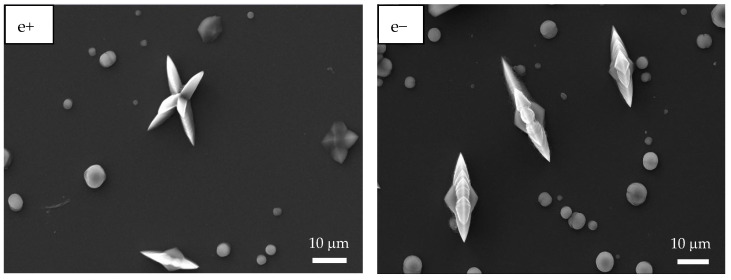
SEM images of CaOx crystals grown via EC on ITO substrate in the presence of homo- and copolymer EPF under positive (+) or negative (−) applied voltage. (**a+**,**a−**) PAA, (**b+**,**b−**), P(PAA-*co*-PSS) 70:30, (**c+**,**c−**) P(PAA-*co*-PSS) 50:50, (**d+**,**d−**) P(PAA-*co*-PSS) 30:70, and (**e+**,**e−**) PSS.

SEM images were consistent with the OM observations, where CaOx formed on ITO in the presence of homo- and copolymer P(AA-*co*-SS) EPF meshes under (+) or (−) applied voltages (Figure 6). As illustrated in Figure 6a+, the presence of spherical crystals with a diameter of approximately 2.0 µm and bipyramidal COD crystals of 10 µm in size were observed when PAA under (+) voltage was utilized. When a (−) voltage was applied, only bipyramidal COD crystals in the size range 2.0–10 µm were observed (Figure 6a−).

When a 70:30 P(PAA-*co*-PSS) mesh was used, few spherical crystals of approximately 2 µm and bipyramidal COD crystals with sharp lateral projections of 5 µm were observed (Figure 6b+). Flower-like COD crystals of approximately 10 µm were also found. Moreover, EPF mesh on the ITO surface was observed after the EC assay, showing a similar behavior to that observed when PAA was utilized (Figure 6b−). For the copolymer EPF samples, Figure 6c+ shows bipyramidal COD crystals, while Figure 6c− shows the presence of spherical crystals and bipyramidal COD crystals with a planar appearance when a 50:50 P(PAA-*co*-PSS) mesh was used. In the case of the 30:70 P(PAA-*co*-PSS) mesh, few spherical crystals and larger flower-like COD crystals of approximately 20 µm were observed (Figure 6d+,d−). Additionally, the presence of an EPF mesh was also noticed. Figure 6e+,e− show spherical crystals around 4.0 µm, bipyramidal COD crystals with elongated sharp lateral projections with dimensions of approximately 12 µm, and flower-like COD crystals of approximately 10 µm when PSS was used.

In addition, SEM-EDS microanalysis of the CaOx crystals and the ITO substrate was performed after the CaOx EC to determine their chemical elemental compositions as we shown in Table 4. We found that the microanalysis of CaOx crystals results of homo- and copolymer P(AA-*co*-SS) EPF meshes on ITO substrate confirmed the presence of the Ca element in all the CaOx crystals and the typical chemical elemental composition of all the EPF meshes was identified, even after 5 min of the EC assay. The EDS analysis of the CaOx highlights the effects of both the polymer composition of the EPF scaffolds and, indirectly, the applied ES voltage, which influences the surface charge of the EPF meshes and, consequently, the CaOx crystallization process. Table 4 shows the presence of the element sulfur (S) and sodium (Na) atoms in the PSS homopolymer and in all P(AA-*co*-SS) copolymers. In addition, the presence of aluminum (Al), silicon (Si), indium (In) and tin (Sn) was identified on the ITO substrate.

**Figure 7:**
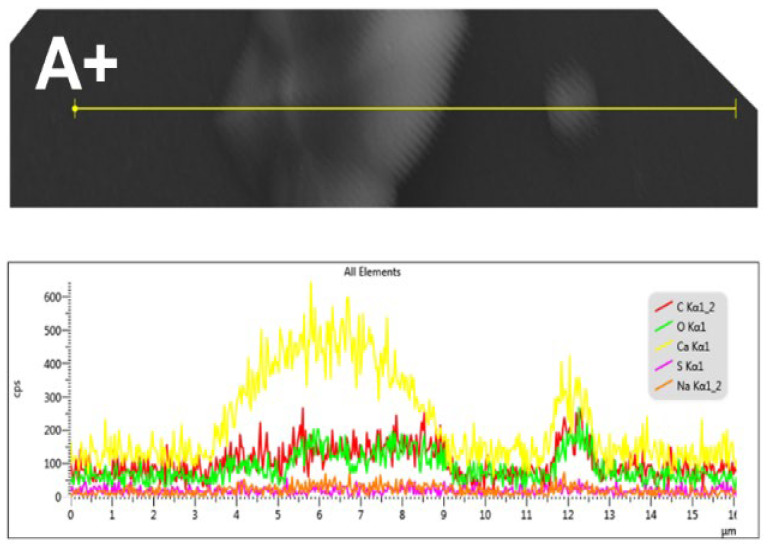
SEM-EDS microanalysis of CaOx grown using PSS EPF meshes under positive (+) applied voltage. Elemental maps show spatial distribution of: carbon (C, red), oxygen (O, green), calcium (Ca, yellow), sulfur (S, fuchsia), and sodium (Na, light red).

Figure 7 shows the results of an SEM-EDS microanalysis of a linear record of the CaOx crystals obtained on the ITO in the presence of PSS when a (+) voltage is applied. This analysis revealed the presence of the carbon (C), oxygen (O) and calcium (Ca) elements from the CaOx, as well as sulfur (S) and sodium (Na) from the PSS.

### 3.4. X-Ray Diffraction

**Figure 8:**
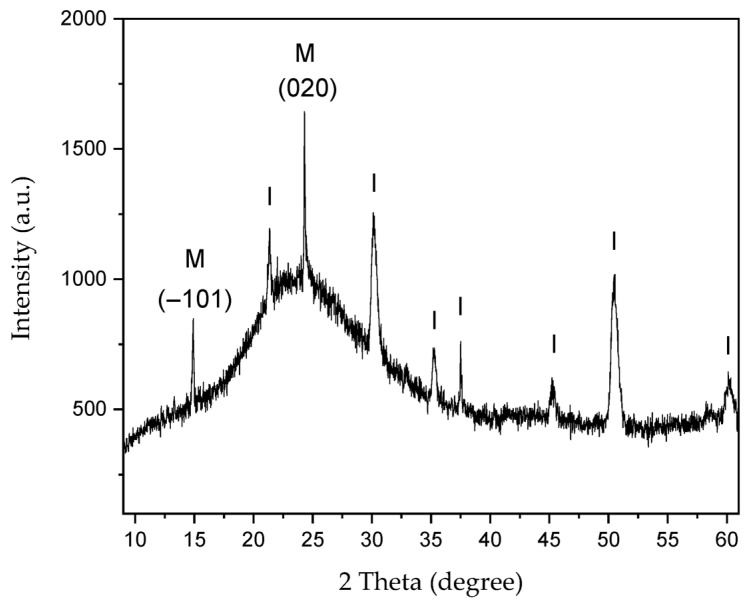
XRD of CaOx crystals grown in control experiment (absence of EPF meshes). Peaks are labeled for: (I) ITO substrate and (M) COM crystalline phase.

**Figure 9:**
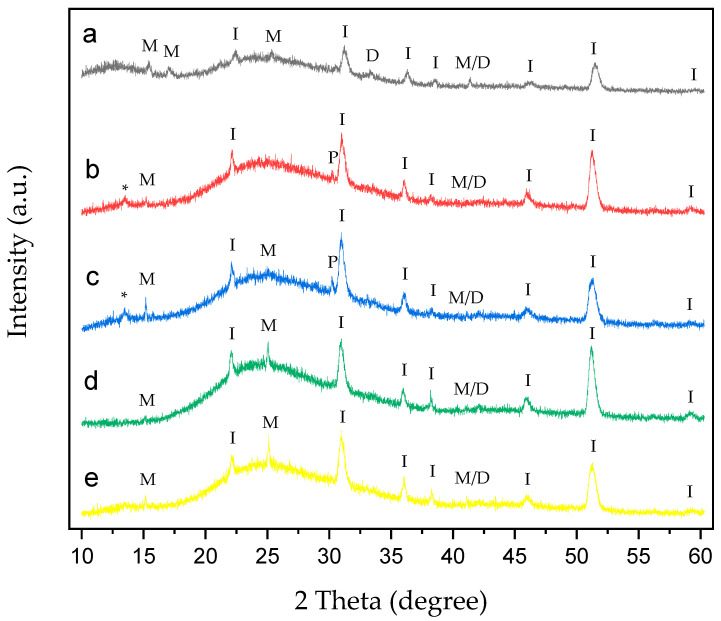
XRD of CaOx crystals grown on ITO with homo- and copolymer EPF under positive (+) applied voltage. (**a**) PAA, (**b**), P(PAA-*co*-PSS) 70:30, (**c**) P(PAA-*co*-PSS) 50:50, (**d**) P(PAA-*co*-PSS) 30:70, and (**e**) PSS. The peak designations of I, P, M, D, M/D and * correspond to the ITO substrate, the adhesive material, COM, COD, COM/COD and unassigned signals, respectively.

**Figure 10:**
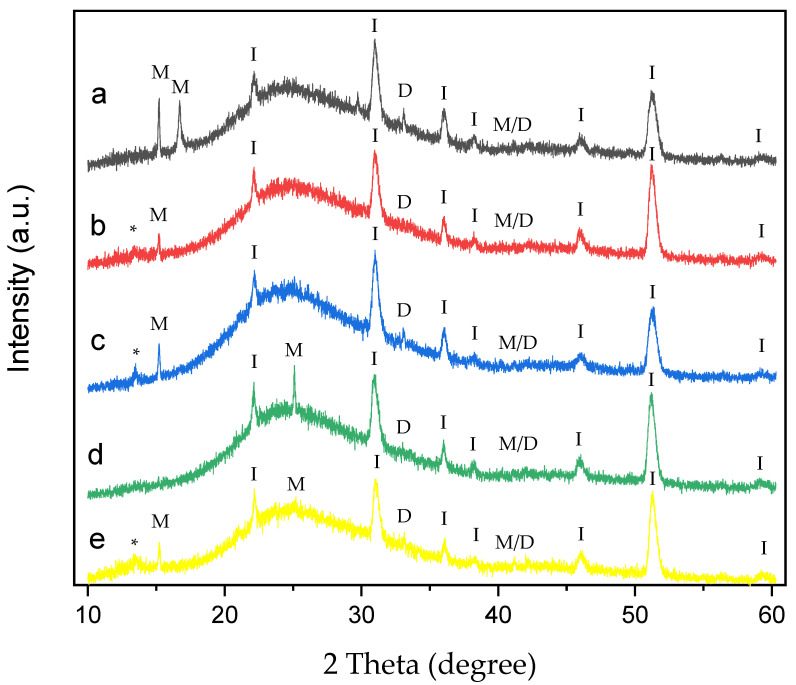
XRD of CaOx crystals grown on ITO with homo- and copolymer EPF under negative (3−) applied voltage. (**a**) PAA, (**b**), P(PAA-*co*-PSS) 70:30, (**c**) P(PAA-*co*-PSS) 50:50, (**d**) P(PAA-*co*-PSS) 30:70, and (**e**) PSS. The peak designations of I, M, D, M/D and * correspond to the ITO substrate, COM, COD, COM/COD and unassigned signals, respectively.

XRD analysis was performed on CaOx crystals grown on an ITO substrate without EPF meshes (Figure 8) and in the presence of homo- and copolymer P(AA-*co*-SS) EPF meshes under (+) or (−) voltages (Figure 9 and Figure 10).

Figure 8 shows the XRD of CaOx crystals grown on the ITO substrate in absence of EPF meshes as control experiment. We observe the 2θ diffraction angles at 2θ = 21.3°, 30.1°, 35.2°, 37.5°, 45.2°, 50.5° and 58.6° for the ITO substrate. It also showed the crystallographic peaks at 2θ = 14.89° and 24.31° for the COM crystal, which correspond to the most intense peaks of the (−101) and (020) planes, respectively. The reference 2θ values and crystallographic planes of the COM and COD crystals were taken from the XRD library of JCPDS cards, being No. 20-231 for COM and No. 17-541 for COD.

XRD patterns of CaOx crystals obtained under (+) (Figure 9) or (−) (Figure 10) applied voltages in the presence of homo- and copolymer EPF showed crystallographic peaks characteristic of the ITO substrate and the COM and COD forms.

Figure 9a shows the XRD of CaOx in the presence of PAA. The crystallographic peaks of ITO can be seen at 2θ = 21.3°, 30.1°, 35.2°, 37.5°, 45.2° and 51°. COM crystallographic peaks are observed at 2θ = 14.4°, 16° and 24.5°, while for COD, peaks are observed at 2θ = 32.6°. Additionally, crystallographic peak at 40.44° belonging to the coexistence of M/D forms was observed. For the 70:30 P(AA-*co*-SS) EPF mesh, the same crystallographic peaks of ITO and only one peak at 2θ = 14.4° for COM were observed (Figure 9b). Here, an unassigned peak was detected at 2θ = 13.4°. XRD pattern of CaOx crystals in the presence of 50:50 P(AA-*co*-SS) EPF mesh show characteristics peaks of ITO (Figure 9c). In the case of 30:70 P(AA-*co*-SS) EPF mesh, similar peaks of ITO and those of COM crystals at 2θ = 14.4° and 24.5° were found (Figure 9d). Figure 9e shows the XRD of CaOx in the presence of PSS. Again, the same crystallographic peaks of ITO as well as that of COM crystals at 2θ = 14.4° and 24.5° were observed, respectively. In addition, the coexistence of M/D forms at 40.44° was in all cases detected.

On the other hand, the XRD of CaOx obtained with PAA under (−) applied voltage shows the crystallographic peaks at 2θ = 14.4°, 16° and 24.5° corresponding to COM, and peaks at 2θ = 32.3° and 2θ = 40.5° assigned to COD crystals and the coexistence of M/D forms, respectively (Figure 10a). In general, XRD of CaOx crystals obtained with all P(AA-*co*-SS) EPF meshes crystallographic peaks of ITO and the coexistence of M/D forms. Moreover, crystallographic peaks associated with pathological plane of COM crystals at 2θ = 14.4° for COM were observed for 70:30 (Figure 10b) and 50:50 (Figure 10c) P(AA-*co*-SS) EPF meshes. The XRD pattern obtained in the presence of the 70:30 P(AA-*co*-SS) EPF mesh showed the characteristic COM peak at 2θ = 14.4° (Figure 10b), while the COD phase was observed at 2θ = 32.4°.

In addition, XRD pattern of CaOx in the presence of 50:50 P(AA-*co*-SS) EPF mesh showed a crystallographic peak at 2θ = 14.4° for COM and also the crystallographic peak of COD at 2θ = 32.4° was observed (Figure 10c). Similarly, in the XRD pattern of CaOx formed using the 30:70 P(AA-*co*-SS) EPF mesh, peaks corresponding to COM and COD were also observed at 2θ = 14.4° and 2θ = 32.4°, respectively. It is worth noting that the crystallographic peak at 2θ = 14.4°, associated with the pathological plane of COM crystals, is absent in this case (Figure 10d). Figure 10e shows the XRD of CaOx obtained in the presence of PSS, where for COM phase at 2θ = 14.4° and 24.5° were observed. Moreover, crystallographic peaks at 2θ = 32.4° and 2θ = 40.5° to COD and of M/D forms were observed, respectively. Again, characteristic crystallographic peaks of ITO were detected.

In addition, the Rietveld method was employed to determine the percentages of COM and COD crystalline phases from the XRD data of CaOx crystals grown on ITO substrates in the presence of homo- and copolymer EPF meshes under positive (+) or negative (−) voltages (Table 5).

Additionally, XRD patterns of homo- and copolymer P(AA-*co*-SS) EPF meshes deposited on the ITO surface applying (+) or (−) voltages (Figures S7 and S8), as well as the XRD of CaOx crystals after EC as polymer films on ITO surface (Figure S9) and in the presence of homo- and copolymer P(AA-*co*-SS) samples used as additive in the ECS (Figure S10). In general, these results show that the XRD of the ITO modified with EPF meshes at (+) or (−) voltage revealed only the crystallographic peaks of the ITO, with values similar to those obtained in the control assay. In the presence of PAA, the 2θ values were 21.4°, 30.3°, 35.3°, 37.6°, 45.2°, 50.6° and 58.5° at (+) voltage (Figure S7) and 2θ = 21.4°, 30.2°, 35.3°, 37.4°, 45.4°, 50.6° and 58.5° at (−) voltage (Figure S8), respectively. Similar crystallographic peaks were found in the XRD patterns of CaOx crystals obtained after EC in the presence of homo- and copolymer P(AA-*co*-SS) films, which were either deposited on an ITO surface (Figure S9) or added to the ECS (Figure S10).

## 4. Discussion

PAA and PSS homopolymers and P(PAA-*co*-PSS) copolymer of different compositions (50:50, 70:30, and 30:70) EPF meshes were prepared and used in CaOx EC assays at (+) or (−) voltage.

FTIR analysis confirmed the presence of PAA and PSS in all copolymer EPF meshes. Furthermore, OM and SEM-EDS analyses demonstrated that the PAA, 50:50 P(PAA-*co*-PSS) and 70:30 P(PAA-*co*-PSS) formed continuous fibers, and that they were easy to handle for the CaOx EC experiments, without the presence of beads. In the case of PSS and 30:70 P(PAA-*co*-PSS) EPF meshes, the presence of beads was identified.

The ES technique offers the possibility of controlling the orientation of the anionic functional groups through the use of (+) or (−) voltage by polarity control of voltage during the ES process. The homopolymers and the P(PAA-*co*-PSS) copolymers contain the anionic groups, which interact with Ca^2+^ ions and can direct the formation of CaOx crystals. Here, the EC was selected because it not only allows more precise control over the mineralization process by enabling the adjustment of parameters such as time, voltage, and current type but also makes it possible to monitor in real time the onset of nucleation through the detection of voltage variations during the EC process, as observed by chronopotentiometric analysis. EC experiments not only enables crystal formation under controlled conditions but also provides valuable insights into the phenomena occurring during the crystallization process, e.g., nucleation stage, which cannot be observed using conventional techniques such as batch crystallization. Therefore, an electrochemical approach using ITO substrates modified with solid EPF meshes as polymeric scaffolds was employed to direct CaOx crystal growth, in contrast to spontaneous crystallization. EC allowed precise control over nucleation kinetics, crystal morphology, and stabilization of specific crystalline phases, thereby better replicating physiological conditions. Moreover, this approach enhanced reproducibility and sensitivity in assessing the influence of polymeric scaffolds on CaOx formation, highlighting its relevance for studying urolithiasis-related processes.

It is known that the anionic functional groups of organic molecules are capable to interact with the Ca^2+^ ions by electrostatic attraction, generating a good nucleation point where the concentration of Ca^2+^ is increased favoring the formation of CaOx crystals [40,66,67]. Indeed, the morphological variation and size of CaOx crystals have an in vivo impact and can influence the crystal-crystal and crystal-cell interaction involved in kidney injury [32,68]. We found that the crystals obtained in the presence of anionic EPF meshes were smaller in size compared to the control tests. A similar result was obtained when the (+) voltage was applied to all copolymer compositions. The decrease in crystal sizes is related to the inhibitory mechanisms described as well as the morphological and structural differences in the surface [16,17].

It is well known that the morphological features of solid organic scaffolds determine the microenvironment in which crystallization occurs, thereby modulating the mineralization process. This study revealed that the morphology of the EPF meshes played a pivotal role in both CaOx nucleation and the stabilization of the crystalline COD and COM phases by shaping the local crystallization microenvironment. EPF meshes composed of continuous, homogeneous fibers from PAA and the P(AA-*co*-SS) 50:50 copolymer produced uniform, bead-free surfaces that promoted controlled crystal growth. This resulted in smaller, more regularly shaped crystals. In contrast, meshes prepared from PSS and the P(AA-*co*-SS) 30:70 copolymer exhibited bead-like defects and surface heterogeneity. This favored localized nucleation, leading to the formation of larger, morphologically diverse crystals. SEM and XRD analyses confirmed that the morphological regularity of the EPF meshes correlated with the stabilization of specific CaOx phases. This study is the first to investigate the CaOx crystallization on EPF meshes prepared from these synthesized copolymers, highlighting the influence of copolymer composition under electrochemical conditions rather than variations in fiber processing parameters.

XRD analysis of CaOx crystals obtained in the presence of homo- and copolymers EPF meshes exhibited similar crystallographic peaks of COM form showing lower intensity peaks with respect to the control experiments under (+) or (−) voltage. Thus, the XRD of CaOx crystals obtained by using PAA and PSS homopolymers stabilized the crystallographic phase of COD at 2θ = 32°. PAA stabilized a crystallographic phase of COM at 2θ = 16° under (+) or (−) voltage. On the other hand, the XRD pattern of CaOx crystals obtained in the presence 50:50 P(PAA-*co*-PSS) showed the crystallographic phase of COD at 2θ = 32° at both voltages. When the 70:30 P(PAA-*co*-PSS) or 30:70 P(PAA-*co*-PSS) EPF meshes were utilized, the XRD patterns of CaOx the crystallographic phase of COD at 2θ = 32° was detected only at (−) voltage. For the COM crystal form, the characteristic COM peak at 2θ = 14.4° was detected when using the 50:50 or 70:30 P(PAA-*co*-PSS) EPF meshes under both positive (+) or negative (−) voltages. In contrast, the XRD pattern of CaOx formed with the 30:70 P(PAA-*co*-PSS) mesh showed stabilization of the COM phase at 2θ = 24.5° under both voltage conditions. Notably, the peak corresponding to the pathological COM phase at 2θ = 14.4° was absent under negative (−) voltage. The obtained XRD patterns of the resulting CaOx crystals suggest that the EPF meshes possess a specific distribution of functional groups capable of stabilizing particular crystalline phases, as demonstrated here using the Rietveld method. Similar observations have been reported for calcium carbonate, where the interatomic distances of functional groups contribute to the stabilization of characteristic crystalline phases [38,69].

The potentiometric curves obtained of the CaOx EC in the presence of homo- copolymer (PAA-*co*-PSS) EPF meshes showed the highest and lowest potential (V) values for PAA and PSS, respectively, while the 50:50 P(AA-*co*-SS) copolymer EPF mesh exhibited intermediate potential (V) values under (+) or (−) voltage. This copolymer EPF mesh generated a lower resistance to the passage of the current by ending with a lower delta than the other two proportions. This is explained because a smaller amount of deposited crystal is not enough to reduce its effects, that is, the type of crystal and its characteristics influence it; therefore, the potentiometric curves allowed to identify the generation of a crystalline deposit on the ITO without discriminating its hydrated form, much less than any of its crystallographic planes.

## 5. Conclusions

We successfully fabricated EPF meshes from PAA and PSS homopolymers and their copolymer P(AA-*co*-SS) using ES under either a positive (+) or negative (−) voltage at room temperature. The results demonstrate that EPF meshes exert a significant influence on the in vitro EC of CaOx. The presence of anionic functional groups in the EPF meshes was critical in modulating crystal morphology, size, and the stabilization of the hydrated CaOx phases (COM and COD). The relative proportions of COM and COD were determined by Rietveld refinement analysis.

These findings establish P(AA-*co*-SS) EPF meshes as versatile and effective platforms for in vitro CaOx crystallization, serving as advanced organic scaffolds for both biomineralization research and pathological crystallization studies. Notably, negatively charged EPF meshes show strong potential as organic matrices for evaluating antiurolithogenic compounds, paving the way for the development of new therapeutic and preventive strategies against urolithiasis.

## Figures and Tables

**Table 1:** The ES parameters employed for the EPF meshes of PAA, PSS homo- and P(AA-*co*-SS) copolymers.

ES Parameters	PAA 8%	P(PAA-*co*-PSS) 70:30	P(PAA-*co*-PSS) 50:50	P(PAA-*co*-PSS) 30:70	PSS 20%
Voltage (+) (kV)	15	15	13	23.5	15
Voltage (−) (kV)	−17	−15	−13	−23.5	−18
Distance (cm)	15	20	16	20	15
Feed rate (µL/h)	800	1200	300	300	1000

**Table 2:** EDS microanalysis of homo- and copolymer P(AA-*co*-SS) EPF meshes on ITO substrate under (+) or (−) voltages.

Condition	C Wt%	O Wt%	S Wt%	Na Wt%	Si Wt%	In Wt%	Al Wt%	Sn Wt%
	V (+)	V (+)	V (+)	V (+)	V (+)	V (+)	V (+)	V (+)
	V (−)	V (−)	V (−)	V (−)	V (−)	V (−)	V (−)	V (−)
PAA	48.0	28.1	-	-	7.8	9.8	2.4	3.9
	41.8	27.8	-	-	10.7	11.5	3.5	4.6
(PAA-*co*-PSS) 70:30	37.5	28.4	1.3	1.4	10.5	12.6	3.4	5.0
	37.1	29.0	1.2	1.4	10.4	12.7	3.3	5.0
(PAA-*co*-PSS) 50:50	51.0	21.5	0.8	1.2	8.5	10.5	2.7	2.0
	42.4	25.0	0.8	1.1	10.1	12.3	3.3	4.9
(PAA-*co*-PSS) 30:70	40.9	25.2	0.2	0.3	13.0	12.2	4.1	1.2
	35.7	28.9	0.6	0.8	12.1	13.3	3.9	2.5
PSS	57.2	17.3	1.1	1.2	7.7	9.2	2.5	3.7
	55.2	17.5	0.4	0.5	8.8	10.5	2.7	4.3

C, O, S, Na, Si, In, Al, and Sn represent carbon, oxygen, sulfur, sodium, silicon, indium, aluminum, and tin detected in the EPF meshes. A dash (-) indicates that the element is absent in the sample. V (+) and V (–) denote positive and negative applied voltages, respectively.

**Table 3:** The variation in the initial and final potential (V) values recorded during EC of CaOx using the EPF meshes and the additives (controls) under (+) or (−) voltages.

Condition	PAA 8%	P(PAA-*co*-PSS) 70:30	P(PAA-*co*-PSS) 50:50	P(PAA-*co*-PSS) 30:70	PSS 20%
Voltage (+)	0.162	0.185	0.095	0.112	0.146
Voltage (−)	0.158	0.214	0.061	0.164	−0.030
Polymer films on ITO	0.085	0.124	0.065	0.126	0.021
Polymer in ECS	−0.028	0.172	0.195	0.157	0.118

**Table 4:** EDS microanalysis of CaOx crystals grown on EPF meshes under (+) or (−) voltages.

Condition	C Wt%	O Wt%	S Wt%	Na Wt%	Ca Wt%	Si Wt%	In Wt%	Al Wt%	Sn Wt%
	V (+)	V (+)	V (+)	V (+)	V (+)	V (+)	V (+)	V (+)	V (+)
	V (−)	V (−)	V (−)	V (−)	V (−)	V (−)	V (−)	V (−)	V (−)
PAA	51.7	21.7	-	-	2.9	9.0	9.9	2.9	2.0
	45.1	36.3	-	-	2.5	2.9	5.6	0.8	1.3
(PAA-*co*-PSS) 70:30	47.9	27.5	0.5	2.8	1.7	6.8	8.9	2.0	1.9
	40.3	33.2	0.1	3.3	3.2	7.0	8.7	2.2	1.8
(PAA-*co*-PSS) 50:50	40.5	29.8	0.3	1.2	2.9	9.0	11.0	2.8	0.3
	36.8	36.3	0.1	2.1	5.2	6.7	8.7	2.1	2.0
(PAA-*co*-PSS) 30:70	36.5	33.5	0.2	1.0	2.9	9.7	11.0	3.1	2.1
	34.2	37.0	0.0	2.8	3.6	7.9	10.0	2.5	1.9
PSS	40.1	27.9	0.0	0.2	3.3	11.0	11.6	3.4	2.5
	36.8	28.5	0.1	0.3	3.8	12.0	12.4	3.7	2.4

C, O, S, Na, Ca, Si, In, Al, and Sn correspond to carbon, oxygen, sulfur, sodium, calcium, silicon, indium, aluminum, and tin present in the EPF meshes. A dash (-) indicates that the element was not detected in the sample, while a zero (0) denotes that the element was detected but not quantified. V (+) and V (−) refer to positive and negative applied voltages, respectively.

**Table 5:** Percentage of COM and COD crystalline phases determined by Rietveld refinement analysis.

ES Parameters	PAA	P(PAA-*co*-PSS) 70:30	P(PAA-*co*-PSS) 50:50	P(PAA-*co*-PSS) 30:70	PSS
Voltage (+) (kV)	COM (98%)	COM (100%)	COM (100%)	COM (100%)	COM (97%)
	COD (2%)	COD (0%)	COD (0%)	COD (0%)	COD (3%)
Voltage (−) (kV)	COM (85%)	COM (54%)	COM (63%)	COM (92%)	COM (90%)
	COD (15%)	COD (46%)	COD (37%)	COD (8%)	COD (10%)

XRD of the CaOx crystals grown in control experiment (absence of EPF meshes) revealed the presence of the COM phase only.

## Data Availability

The original contributions presented in this study are included in the article/Supplementary Material. Further inquiries can be directed to the corresponding author.

## Supplementary Materials

The following are available online at https://www.mdpi.com/article/10.3390/polym17212888/s1, Figure S1: MO images of electrospun nanofiber (EPF) meshes produced from PAA, PSS and PAA:PSS under both positive (+) or negative (−) applied voltage, Figure S2: OM images of CaOx crystals grown via EC on ITO substrate in the presence of homo- and copolymer EPF under positive (+) or negative (−) applied voltage, Figure S3: FTIR spectra of electrospun nanofiber (EPF) produced from homopolymer under both positive (+) or negative (−) applied voltage, Figure S4: FTIR spectra of electrospun nanofiber (EPF) produced from copolymer under positive (+) or negative (−) applied voltage, Figure S5: Chronamperometry curves for EC of CaOx in the presence of homo- and P(AA-*co*-SS) copolymer using as polymer film additive on the ITO, Figure S6: Chronamperometry curves for EC of CaOx in the presence of homo- and P(AA-*co*-SS) copolymer using as additive in ECS, Figure S7: XRD spectra of electrospun nanofiber (EPF) produced from homo- and copolymer under positive (+) voltage on the ITO, Figure S8: XRD spectra of electrospun nanofiber (EPF) produced from homo- and copolymer under negative (−) voltage on the ITO, Figure S9: XRD spectra of CaOx crystals grown via EC on ITO substrates in the presence of homo- and copolymer EPF using as additive on the ITO, Figure S10: XRD spectra of CaOx crystals grown via EC on ITO substrates in the presence of homo- and copolymer EPF using as film on the ITO.
