# Supplementary material for: Electrocrystallization of Calcium Oxalate Mediated by Electrospun Polymer Fiber Using Poly(acrylic acid-co-4-styrene sulfonate)
Source: Polymers (Basel). 2025 Oct 29;17(21):2888. doi: 10.3390/polym17212888 (PMC12610098; doi:10.3390/polym17212888)
Supplement: Supplementary file 1 [file polymers-17-02888-s001.zip › polymers-3887856-supplementary.pdf]

Electrocrystallization of Calcium Oxalate Mediated by Electrospun Polymer Fiber Using Poly(acrylic acid-*co*-4-styrene sulfonate)

Andrónico Neira-Carrillo [1,*], Eddie Nieto [1], Nicole Butto-Miranda [1], Dania Cataldo [1], Bruno F. Urbano [2] and Mehrdad Yazdani-Pedram [3]

[1] Department of Biological and Animal Sciences, Faculty of Veterinary and Animal Sciences, University of Chile, Santiago 8820808, Chile; eddienieto.g@gmail.com (E.N.); nbutto@veterinaria.uchile.cl (N.B.-M.); dania.cataldo2@gmail.com (D.C.)

[2] Department of Polymer Chemistry, Faculty of Chemical Science, University of Concepción, Concepción 3349001, Chile; burbano@udec.cl

[3] Department of Organic and Physical Chemistry, University of Chile, Santiago 8380544, Chile; myazdani@uchile.cl

* Correspondence: aneira@uchile.cl; Tel.: +562-29785674

Supplementary Materials

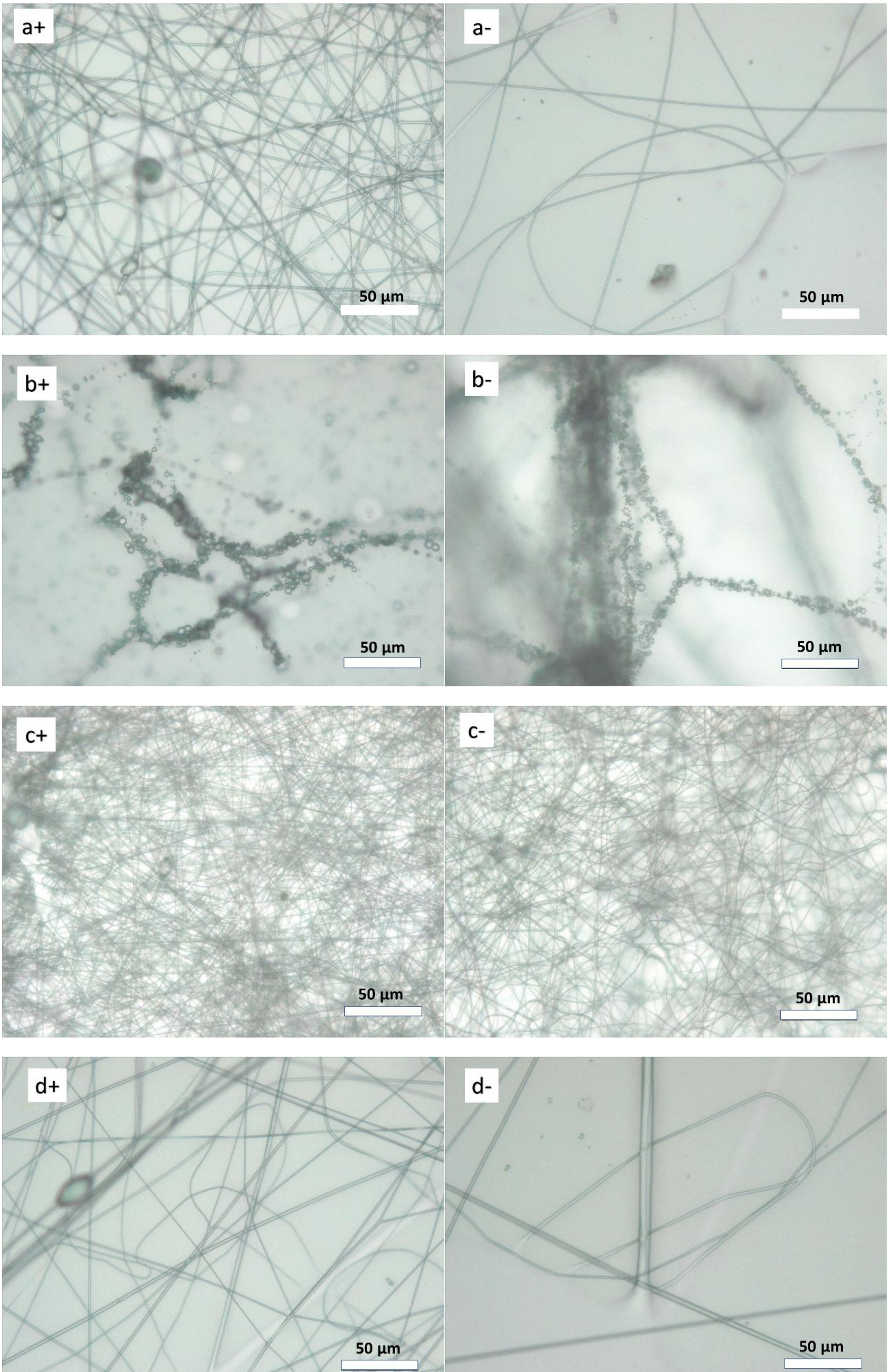
a+
50 µm
a-
50 µm
b+
50 µm
b-
50 µm
c+
50 µm
c-
50 µm
d+
50 µm
d-
50 µm

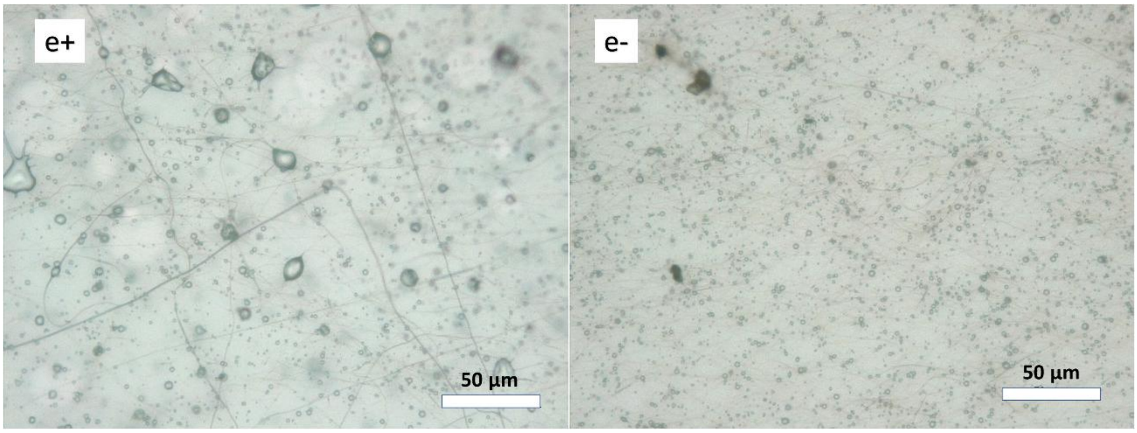

Figure S1. OM images of homo- and copolymer EPF meshes under positive (+) or negative (-) applied voltage: a) PAA, b) PSS, c) P(PAA-*co*-PSS) 50:50, d) P(PAA-*co*-PSS) 70:30, and e) P(PAA-*co*-PSS) 30:70. Optical magnifications were 40x.

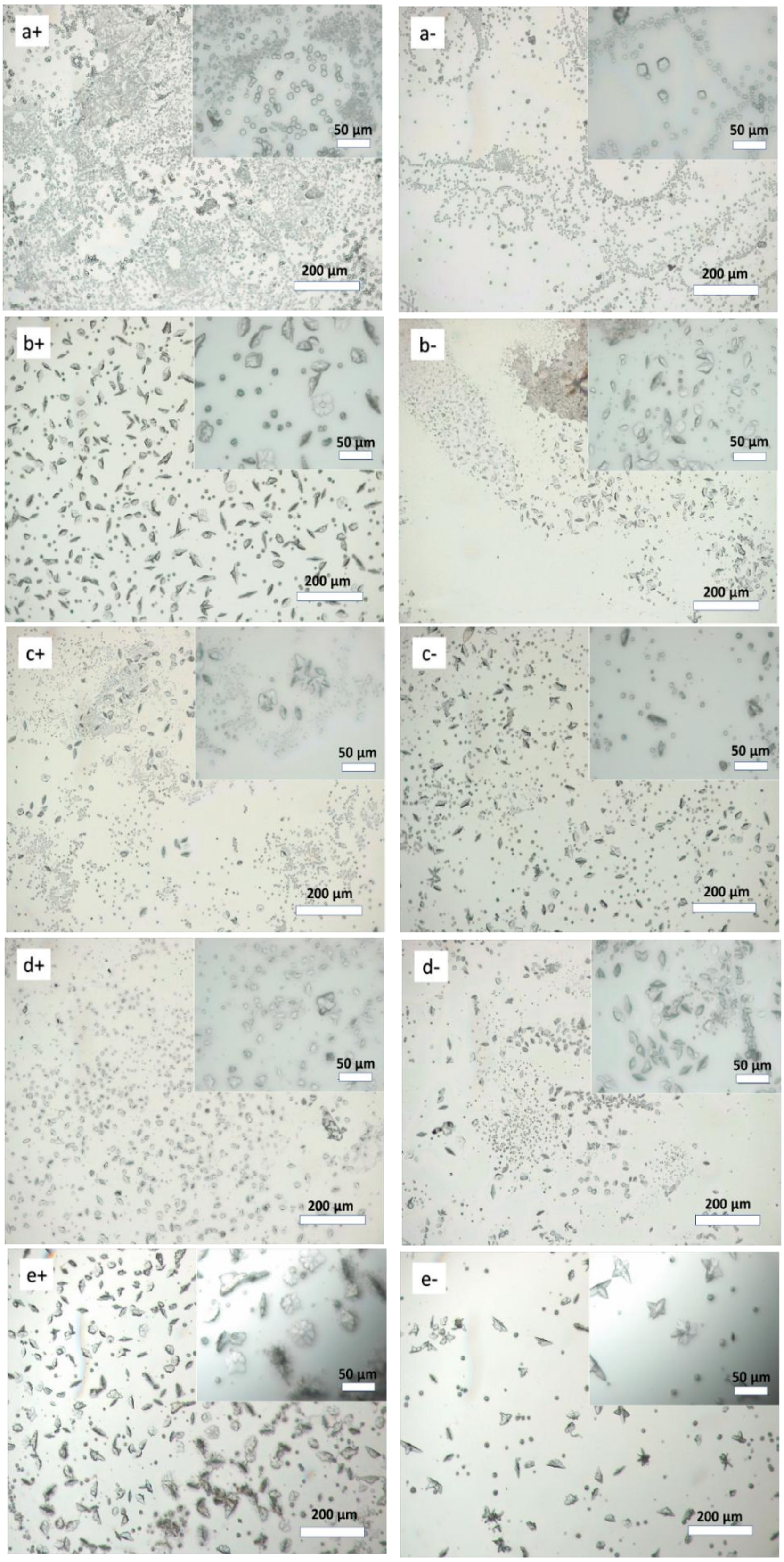

Figure S2. OM images of CaOx crystals grown via EC on ITO substrate in the presence of homo- and copolymer EPF under positive (+) or negative (-) applied voltage. a) PAA, b) PSS, c) P(PAA-*co*-PSS) 50:50, d) P(PAA-*co*-PSS) 70:30, e) P(PAA-co-PSS) 30:70. Optical magnifications were 10x and 40x.

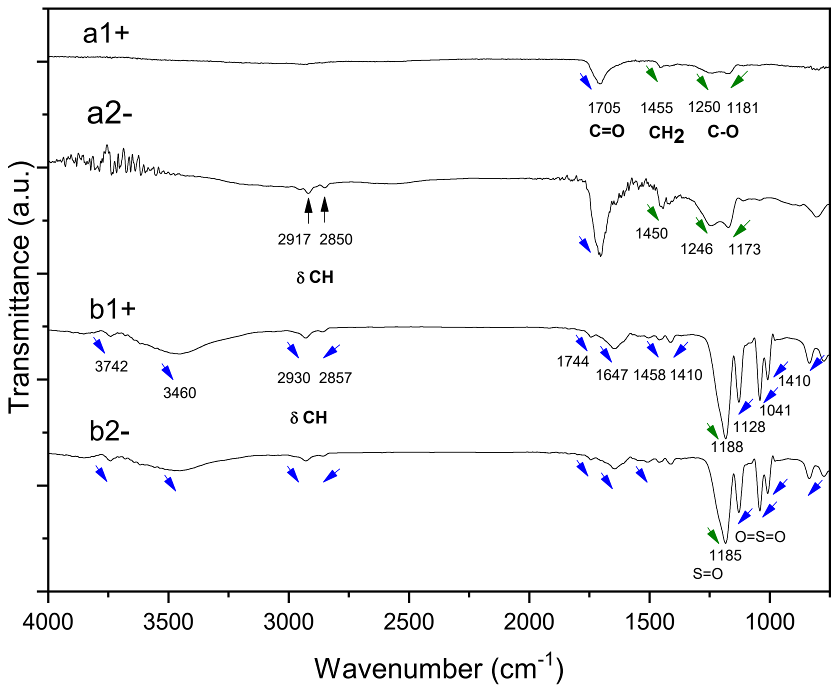

Figure S3. FTIR spectra of homopolymer EPF meshes produced under positive (+) or negative (-) applied voltage. a1) PAA +, a2) PAA -, b1) PSS +, and b2) PSS -. The green and blue arrows indicate the presence and absence of shifts in the position of the absorption bands between the two FTIR spectra at both voltages.

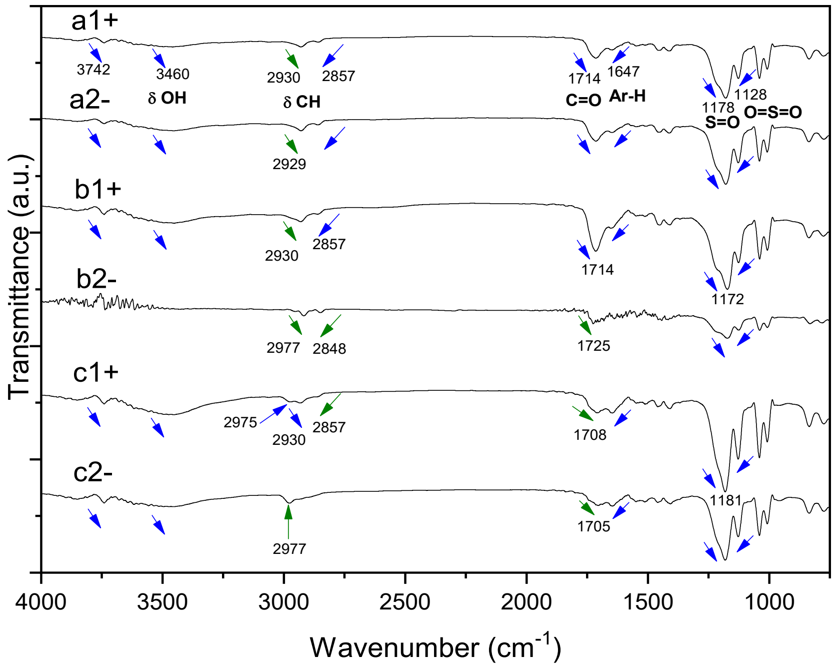

Figure S4. FTIR spectra of P(PAA-*co*-PSS) copolymer EPF meshes produced under positive (+) or negative (-) applied voltage. a1) P(PAA-*co*-PSS) 50:50 +, a2) P(PAA-*co*-PSS) 50:50 -, b1) P(PAA-*co*-PSS) 70:30 +, b2) P(PAA-*co*-PSS) 70:30 -, c1) P(PAA-*co*-PSS) 30:70 +, and c2) P(PAA-*co*-PSS) 30:70 -. The green and blue arrows indicate the presence and absence of shifts in the position of the absorption bands between the two FTIR spectra at both voltages.

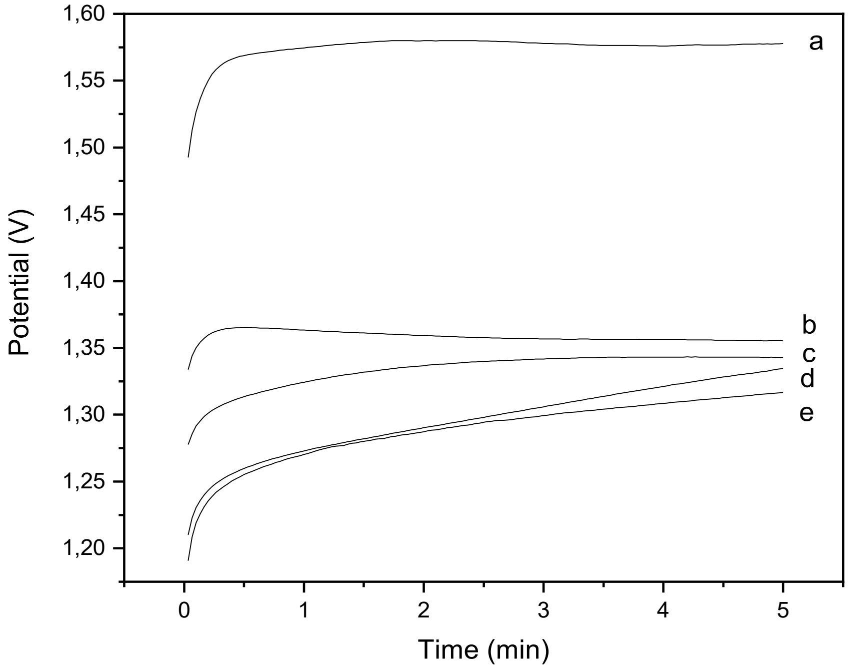

Figure S5. Chronamperometry curves for EC of CaOx in the presence of homo- and copolymer P(AA-*co*-SS) using as polymer film additive on the ITO. a) PAA, b) PSS, c) 50:50 P(PAA-*co*-PSS), d) 70:30 P(PAA-*co*-PSS), and e) 30:70 P(PAA-*co*-PSS).

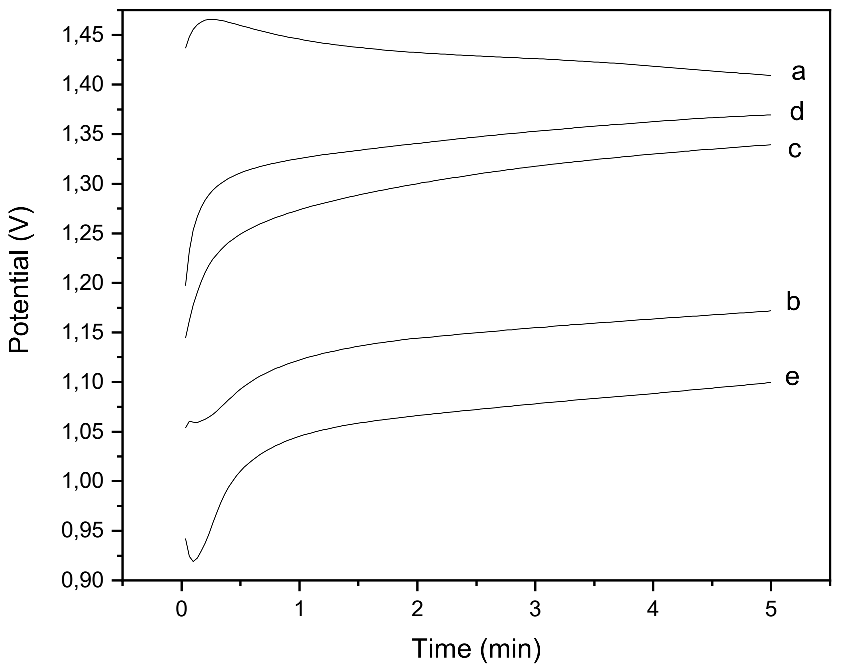

Figure S6. Chronamperometry curves for EC of CaOx in the presence of homo- and copolymer P(AA-*co*-SS) using as additive in ECS. a) PAA, b) PSS, c) 50:50 P(PAA-*co*-PSS), d) 70:30 P(PAA-*co*-PSS), and e) 30:70 P(PAA-*co*-PSS).

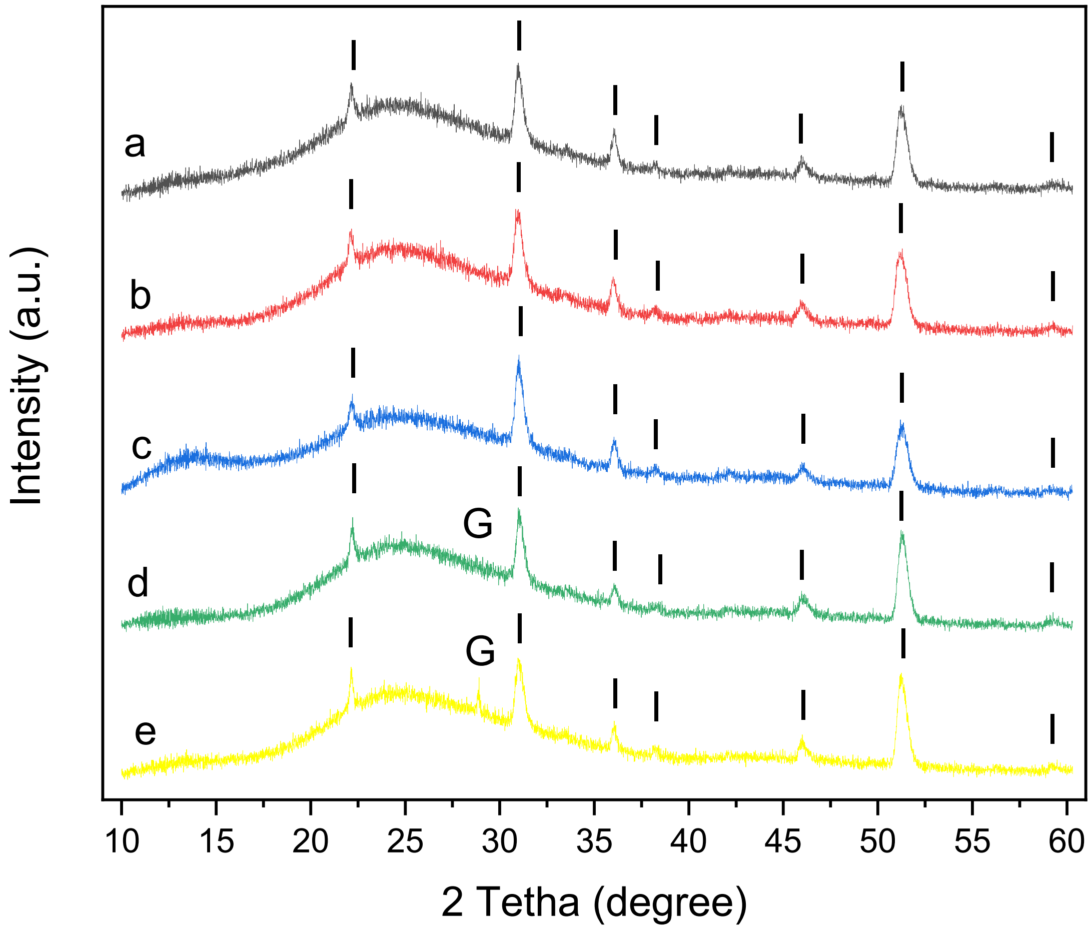

Figure S7. XRD of the P(PAA-*co*-PSS) copolymer EPF meshes produced under positive (+) applied voltage on ITO. A) PAA, B) PSS, C) 50:50 P(PAA-*co*-PSS), D) 70:30 P(PAA-*co*-PSS), and E) 30:70 P(PAA-*co*-PSS). The peak designations of I and G correspond to the ITO substrate and the graphitic material, respectively.

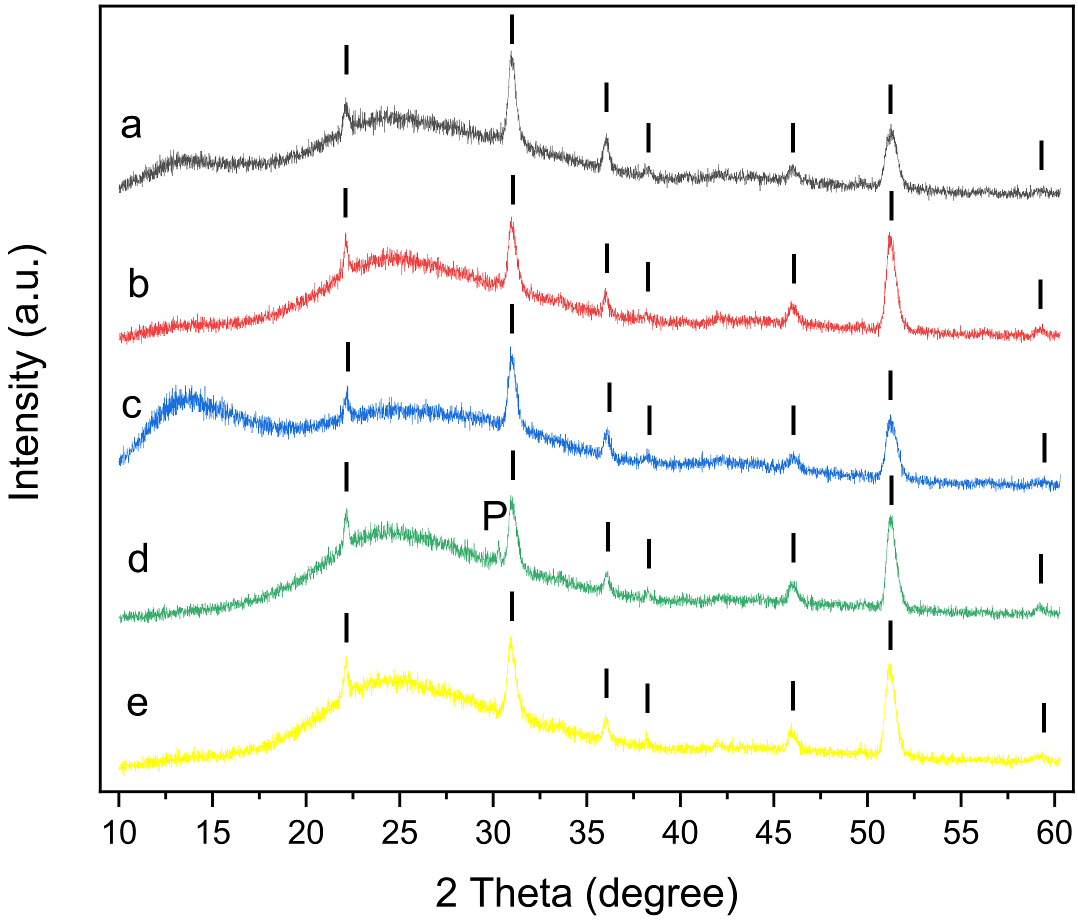

Figure S8. XRD of homo- and copolymer EPF meshes under negative (-) applied voltage on ITO. A) PAA, B) PSS, C) 50:50 P(PAA-*co*-PSS), D) 70:30 P(PAA-*co*-PSS), and E) 30:70 P(PAA-*co*-PSS). The peak designations of I and P correspond to the ITO substrate and the adhesive material, respectively.

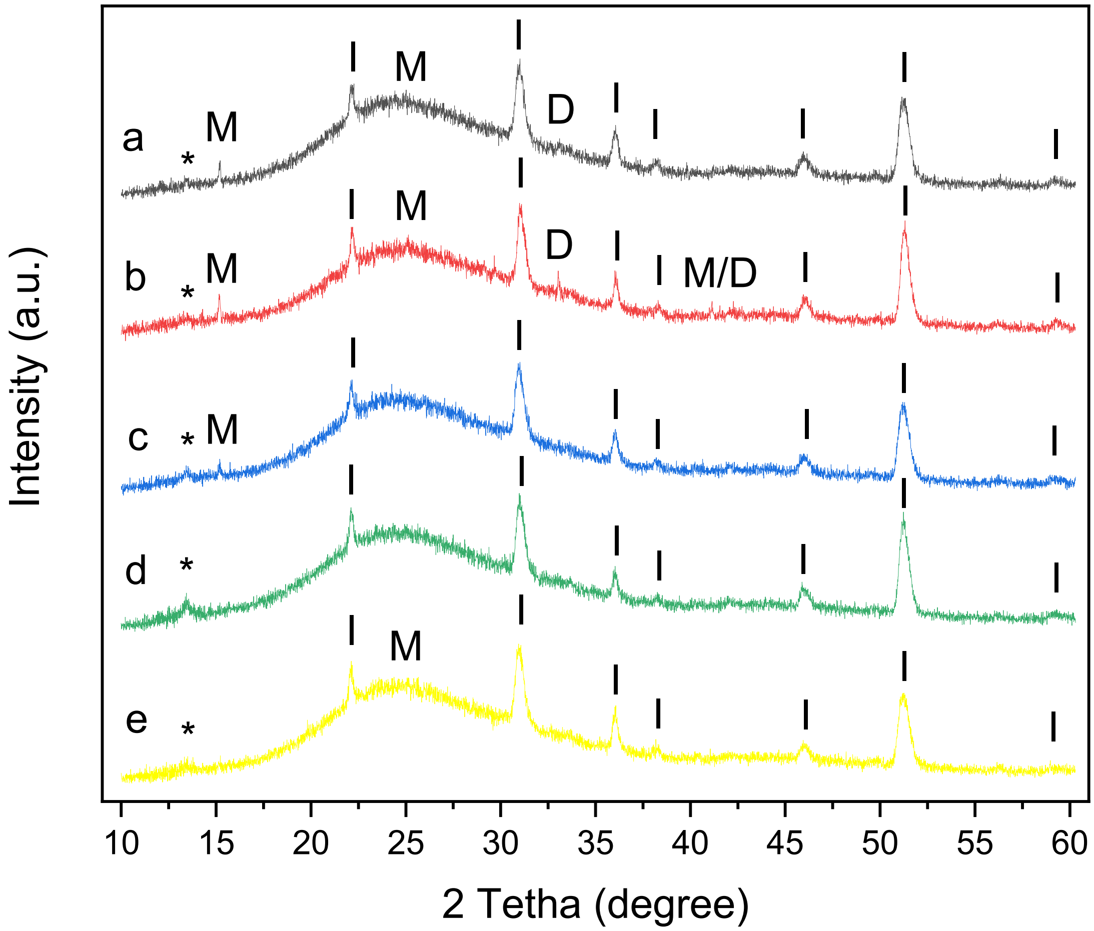

Figure S9. XRD of CaOx crystals grown via EC on ITO substrates in the presence of homo- and copolymer EPF using as film on the ITO. A) PAA, B) PSS, C) 50:50 P(PAA-*co*-PSS), D) 70:30 P(PAA-*co*-PSS), and E) 30:70 P(PAA-*co*-PSS). The peak designations of I, M, D, M/D and * correspond to the ITO substrate, COM, COD, COM/COD and unassigned signals, respectively.

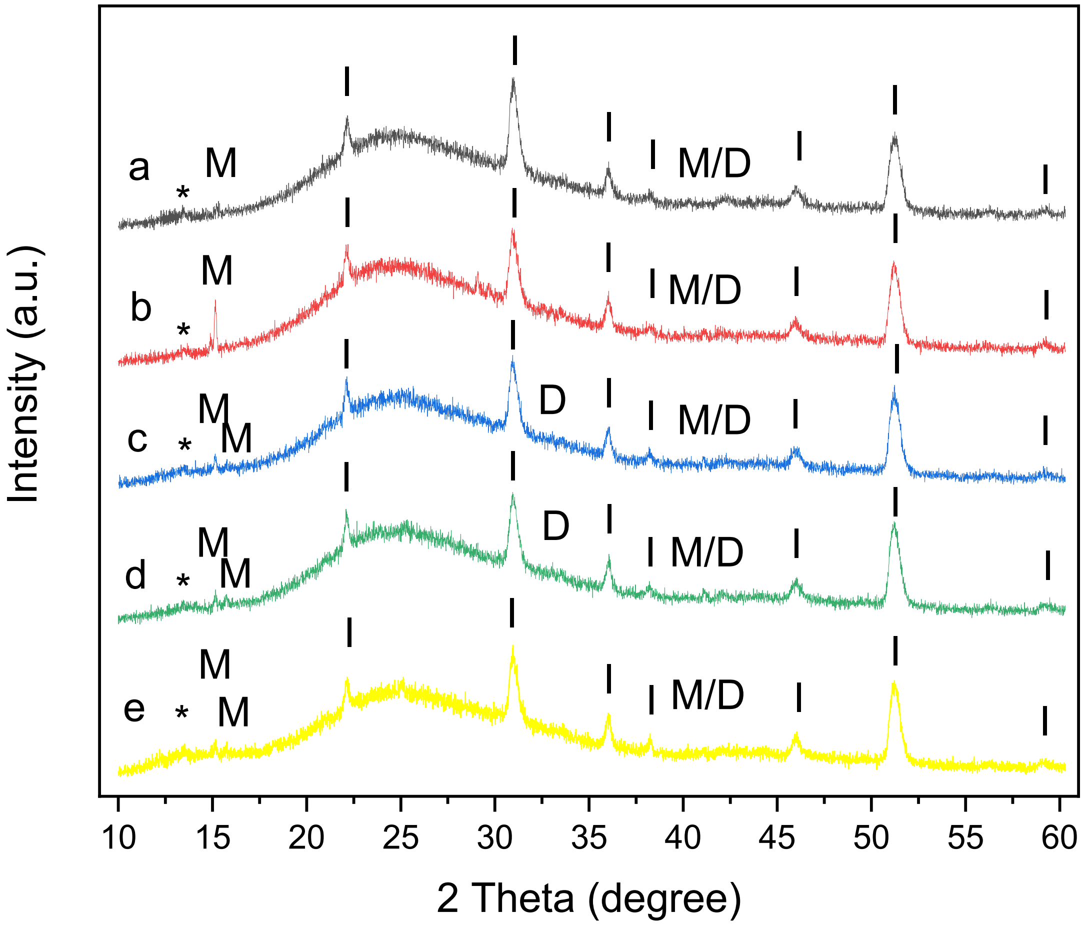

Figure S10. XRD of CaOx crystals grown via EC on ITO substrates in the presence of homo- and copolymer EPF using as additive in the ECS. A) PAA, B) PSS, C) P(PAA-*co*-PSS) 50:50 P(PAA-*co*-PSS), D) P(PAA-*co*-PSS) 70:30, and E) P(PAA-*co*-PSS) 30:70. The peak designations of I, M, D, M/D and * correspond to the ITO substrate, COM, COD, COM/COD and unassigned signals, respectively.
